# Effects of Aerobic Exercise on the Pulmonary Functions, Respiratory Symptoms and Psychological Status of People Living With HIV

**Published:** 2016-02-27

**Authors:** Happiness Anulika Aweto, Ayoola Ibifubara Aiyegbusi, Adaora Justina Ugonabo, Titilope Adenike Adeyemo

**Affiliations:** ^a^ Department of Physiotherapy, College of Medicine, University of Lagos, PMB 12003, Idi-Araba, Lagos, Nigeria; ^b^ Department of Haematology and Blood transfusion, College of Medicine, University of Lagos, PMB 12003, Idi-Araba, Lagos, Nigeria

**Keywords:** Aerobic exercise, Pulmonary, Psychological status, HIV

## Abstract

**Background:** Pulmonary complications, respiratory symptoms and depression are common occurrences which contribute to the morbidity and mortality seen in individuals living with HIV/AIDS. This study investigated the effect of aerobic exercise on the pulmonary functions, respiratory symptoms and psychological status of people living with HIV.

**Methods:** This study was conducted in Lagos, Nigeria from October 2014 to May 2015. Forty eligible individuals with HIV aged 18 yr and above participated, of which 33 cooperated to the end. They were recruited from the HIV/AIDS Prevention and Intervention Initiative (APIN) Clinic, Lagos University Teaching Hospital, Nigeria and were randomly assigned to either the study or the control group. The study group received aerobic exercise training three times a week for six weeks and counselling while the control group received only counselling. Pulmonary functions, respiratory symptoms and psychological status were evaluated at baseline and at six weeks. Inferential statistics of paired and independent t-test were used to analyze the data.

**Results:** Comparison of mean changes in the pulmonary variables of the study group with those of the control group showed significant differences in all but in the respiratory rate (RR) - [Forced Expiratory Volume in one second: ,*P*=0.001, Forced Vital Capacity: *P*=0.001, Peak Expiratory Flow: *P*=0.001]. There were also significant differences between the mean changes in respiratory symptoms (*P*=0.001) and depressive symptoms (*P*=0.001) of study group and those of the control group.

**Conclusions:** Aerobic exercise training significantly improved pulmonary functions as well as significantly reduced respiratory and depressive symptoms in people living with HIV.

## Introduction

Pulmonary complications such as *Pneumocystis carinii* pneumonia, tuberculosis, bacterial pneumonia and neoplasms have been a major cause of morbidity and mortality in patients with HIV infection^1,2^. Respiratory symptoms which result from a wide spectrum of pulmonary complications are frequent complaints among HIV-infected individuals and may be HIV related or non-HIV related^2^. These symptoms which include cough, sputum production, wheeze, dyspnea, and pleuritic chest pain can either present alone or in combination^3^. HIV infection is an independent risk factor for reduced pulmonary function particularly in individuals whose CD4 count is reduced and manifests clinically with increased respiratory symptoms^4^. Low CD4 count (<350 cells/mm^3^) were independently associated with *Mycobacterium tuberculosis*/HIV co-infection^5^. Reduced pulmonary function is associated with impaired ventilation which progressively worsens over time and could result in life threatening conditions like atelectasis, hypoxia, respiratory failure and cardiovascular mortality^6^. Antiretroviral (ARV) therapy has also been shown as an independent predictor of increased airway obstruction and reduced pulmonary function^1^.

Depression is amongst the most common neuropsychiatric disorders that affect individuals with HIV infection^7^. It can be a consequence of HIV-induced brain injury, shock after diagnosis, appearance of symptoms, death of another HIV patient as well as side effect of ARV medication^8^.Depression has been associated with immune system malfunctioning and with poorer disease outcomes in patients. Although it can be effectively treated in most individuals, less than one half of patients with depression are correctly diagnosed and still fewer receive adequate treatment^9^. Depression is associated with non-adherence to ARV therapy, progression of HIV disease, and decline in CD4 cell count^10^.

Although exercise has been effective and safe for HIV infected population, 55.4% to 73% of people living with HIV are physically inactive^11,12^. Exercise has consistently been listed as one of the most popular self-care non-pharmacological therapies but few studies have evaluated the effects of exercise on some common self-reported symptoms^13^.

This study investigated the effects of aerobic exercise on pulmonary functions, respiratory symptoms and depression in people living with HIV.

## Methods

This study was conducted from October 2014 to May 2015. Fifty-three people living with HIV were recruited through referrals by physicians from the HIV/AIDS Prevention and Intervention Initiative (APIN) Clinic, Lagos University Teaching Hospital, Idi-Araba, Lagos. Forty-seven of them agreed to participate in the study while six declined after the requirements of the study were explained to them.

They were screened for eligibility based on the inclusion and exclusion criteria of the study. Forty were included as seven were excluded based on the criteria that they were people living with HIV who: were 18 yr and above and on current use of Highly Active Anti-Retroviral Therapy (HAART) as well as met Centre for Disease Control and Prevention (CDC) classifications A_2_ (Asymptomatic, non-AIDS) or B_2_ (Symptomatic, non-AIDS)^14^. Those who had current active opportunistic infections such as tuberculosis, history of cardiovascular disease (screened using American Heart Association- American College of Sports Medicine Participation Screening Questionnaire), significant cognitive impairment as well as those who were pregnant or involved in a regular exercise program were all excluded. The subjects who met the inclusion criteria signed the informed consent form and were then randomly assigned equally into study and control groups (20 in each).

**Figure 1:**
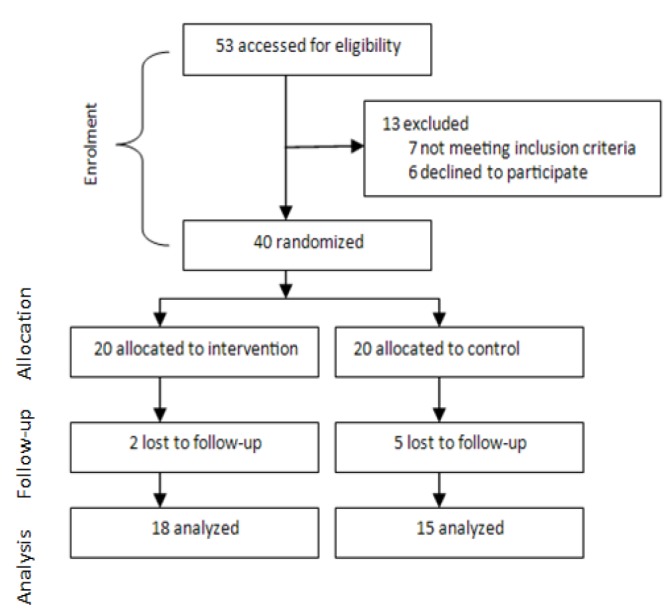
Flowchart of progress through the study

The randomization was done by simple random sampling using the fish bowl method where numbers one to forty were written on cards, then wrapped and put into a container. Numbers one to twenty represented the study group while numbers 21 to 40 represented the control group. Eligible subjects picked the number from the container. Two subjects from the study group and five from the control group withdrew before the end of the study for reasons such as inadequate time to participate and the need to travel out of the state (Figure 1).

Ethical approval was obtained prior to the study from the Health Research and Ethics Committee of the Hospital. The clinical trial registration number is PACTR201602001465776.

### Procedure

The baseline measurements of all the selected pulmonary outcome parameters were taken for all the subjects after a rest period of 15 min prior to the intervention. Forced Vital Capacity (FVC), Forced Expiratory Volume in one second (FEV_1_) and Peak expiratory flow rate (PEFR) were measured using a spirometer (CONTEC SP10, Model No: JE1405100271, China). St George’s Respiratory Questionnaires (SGRQ) and Becks Depression Inventory (BDI) were also filled out by the subjects at baseline. These are measures of respiratory symptoms and depression respectively. SGRQ is a standardized self-administered airways disease-specific questionnaire^15^. It contains 50 items (covering 76 levels) divided into three sub-scales: "Symptoms" (8 items), including several respiratory symptoms, their frequency and severity; "Activity" (16 items), is concerned with activities that cause or are limited by breathlessness; and "Impacts" (26 items), which covers a range of aspects concerned with social functioning and psychological disturbances resulting from airways disease. The BDI is a self-report depressive symptom index that includes 21 items. Participants rated each symptom on severity and individual items are scored from 0 to 3. Total scores range from 0 to 63, and a score of 9 is thought to be indicative of at least mild depression. A score of 15 is designated as a possible depression cut-off in the general population^16^. Concurrent validity has been demonstrated with other measures of depression, internal consistency, and instrument stability^16^.

The study group underwent aerobic exercise using bicycle ergometer (Sunbrahrer, Model No: TZ-4102) and counselling sessions. Aerobic exercise on bicycle ergometer was preceded by a 5 min warm up exercise and ended with a 5 min cool down exercise. They rode on the stationary bike with gradual exercise loads administered incrementally based on 50-60% heart rate reserve (moderate intensity). The exercise duration and frequency was 30 min, 3 days a week. Progression was done by gradually increasing the resistance by 5 watts after every week such that by the 6^th^ week, the subjects exercised at 30 watts power. The counselling sessions were for 30 min, once in two week. The control group had no therapeutic exercise intervention but had group counselling sessions for 30 min, once in two week. Measurements of all the selected outcome parameters were also taken for all the subjects at the end of the 6^th^ week.

#### Data Analysis

Data were analyzed using the SPSS version 20 (Chicago, IL, USA). Descriptive statistics of mean, standard deviation and percentages were used to summarize data. Inferential statistics of paired *t*-test and independent *t*-test were used to compare data within group and between groups respectively. Significant level was set at *P*<0.05.

## Results

Forty eligible individuals (25 females and 15 males) with HIV started the study but 33 of them (23 females and 10 males) completed it. The study group consisted of 18 subjects (55%) and the control group 15 subjects (45%) at the end of the study. The age range for the study group was 20 yr to 45 yr while that of the control group was 24 yr to 41 yr. The mean ages of the study and control groups were 30.67 ±5.83 yr and 32.07 ±5.36 yr, respectively. The mean body mass index (BMI) were 25.40 ±1.74 kg/m^2^ and 26.09 ±1.37 kg/m^2^, respectively. Comparison of the baseline data of the study group with that of the control group revealed that there was no significant difference (Table 1). This shows that subjects in the two groups were similar and comparable.

**Table 1:** Comparison between baseline data of the study and control groups

**Variables**	**Control group**		**Intervention group**		***P*** ** value**
	**Mean**	**SD**	**Mean**	**SD**	
Age (yr)	32.07	5.36	30.67	5.83	0.482
Body mass index (BMI) (kg/m^2^)	26.09	1.37	25.40	1.74	0.211
Forced Vital Capacity ((litres)	1.72	0.73	1.99	0.75	0.314
Forced Expiratory Flow in one sec ((litres)	1.23	0.52	1.32	0.61	0.650
Peak Expiratory Flow (litres/min)	1.47	0.68	1.70	1.02	0.452
Respiratory Rate (breaths/min)	19.13	2.33	19.44	2.67	0.732
St George Respiratory Questionnaire	29.30	15.53	28.75	20.82	0.630
Becks Depression Inventory	10.06	5.96	10.33	6.48	0.901

### Pulmonary function result

With the exception of the respiratory rate (RR), paired *t*-test comparison between the pre-test and post-test pulmonary outcome mean values of the study group showed that there were significant improvements (FEV_1_: *P*=0.001, FVC: *P*=0.001, PEF: *P*=0.001) while no significant improvements were observed in those of the control group (Table 2).

**Table 2:** Comparison of pre-test and post-test pulmonary variable mean values within and between the control and intervention groups

**Pulmonary Variables**	**Control group**			**Intervention group**			
	**Before**	**After**	**Within**	**Before**	**After**	**Within**	**Between**
	**Mean (SD)**	**Mean (SD)**	***P*** ** value**	**Mean (SD)**	**Mean (SD)**	***P*** ** value**	***P*** ** value**
Forced Vital Capacity (l)	1.72 (0.73)	1.66 (0.67)	0.302	1.99 (0.75)	2.70 (0.65)	0.001	0.001
Forced Expiratory Flow in one sec (l)	1.23 (0.52)	1.20 (0.53)	0.501	1.32 (0.65)	1.98 (0.55)	0.001	0.001
Peak Expiratory Flow (l/min)	1.47 (0.68)	1.58 (0.74)	0.841	1.70 (1.08)	2.53 (0.79)	0.001	0.001
Respiratory Rate (breaths/min)	19.13 (2.02)	19.83 (2.04)	0.833	19.44 (2.67)	19.06 (1.66)	0.670	0.490

Comparison of changes in the pulmonary outcome variables of the study group with those of the control group showed significant differences in all but in RR - [FEV_1_: *P*=0.001, FVC: *P*=0.001, PEF: *P*=0.001] (Table 2).

### Respiratory symptoms result

Comparisons between the pre-test and post-test respiratory symptoms mean scores of the study group also showed that there was significant improvement – (SGRQ: *P*=0.001) while that of the control group showed no significant improvement (Table 3). Comparisons of the change in the respiratory symptoms mean score of the study group with those of subjects in the control group showed a significant difference (*P*=0.001) (Table 3).

**Table 3:** Comparison of pre-test and post-test mean respiratory symptoms and depression scores within and between the control and intervention groups

**Variables**	**Control group**			**Intervention group**			
	**Before**	**After**	**Within**	**Before**	**After**	**Within**	**Between**
	**Mean (SD)**	**Mean(SD)**	***P*** ** value**	**Mean (SD)**	**Mean (SD)**	***P*** ** value**	***P*** ** value**
St George Respiratory Questionnaire (SGRQ)	29.30 (15.53)	31.25 (13.89)	0.064	28.75 (20.83)	18.71 (12.51)	0.001	0.001
Becks Depression Inventory (BDI)	10.06 (5.96)	8.33 (5.80)	0.852	10.33 (6.48)	3.50 (1.27)	0.001	0.001

### Result of Evaluation of Depression

Comparisons between the pre-test and post-test depression mean score of the study group also showed that there was significant improvement (BDI: *P*=0.001) while that of the control group showed no significant improvement (Table 3).

Comparisons of the change in the depression mean score of the study group with those of subjects in the control group also showed a significant difference (*P*=0.001) (Table 3).

## Discussion

The aim of this study was to determine the effects of aerobic exercise on the pulmonary functions, respiratory symptoms and psychological status of people living with HIV. The results of the study showed that after 6 weeks of the study there were significant improvements the pulmonary functions, respiratory and depressive symptoms of subjects in the study group while there were no significant improvements in those of the control group. With the exception of RR, comparison of the mean changes in the pulmonary outcome variables, respiratory and depressive symptoms of subjects in the study group with those of the control group showed significant differences.

The finding that the pulmonary outcome variables significantly improved in the study group implies that six weeks aerobic exercise conducted for 30 min three times a week can lead to an appreciable improvement in pulmonary functions of people living with HIV. This is asserted because both the study and control groups had similar baseline characteristics at the beginning of the study. The reason for the improvement may be due to the long-term effect of aerobic exercise which leads to an expansion of the oxygen transport system reflected by the augmented capacity for maximal work^17^.There is larger lung size and vital capacity, higher blood volume and total hemoglobin, larger stroke volume, maximal oxygen uptake and arterio-venous oxygen difference^17^. Physical activity improved pulmonary function in healthy sedentary adults^18^. Aerobic exercise could improve pulmonary function and alter exercise breathing pattern in children^19^. Tehrani et al.^20^ showed significant improvement of pulmonary function in obese Iranian females after aerobic exercise training. Besides, a positive relationship existed between aerobic training and pulmonary function among young adults^21^.

However, although there was a slight reduction in respiratory rate of subjects in the study group post intervention, this reduction was not statistically significant. This could be due to the fact that 6 weeks duration of aerobic training was not long enough to bring about an appreciable reduction in respiratory rate. Incidentally, we did not come across any study that assessed the effect of aerobic exercise on respiratory rate of individuals except for one study that reported a significant decrease in respiratory rate in adolescents after 45 days of yoga training^22^.

The significant reduction in respiratory symptoms of the study group compared with the control group indicates that aerobic exercise reduced respiratory symptoms in people living with HIV. This corroborates the report of prior studies on the effect of physical activity on some other respiratory conditions. Goncalves et al.^23^ reported a reduction in the respiratory symptoms in individuals with moderate to severe / persistent asthma after three months of physical activity. Aerobic exercise and strength training significantly reduced respiratory symptoms in patients with COPD^24^.

In addition to the negative physical and psychological changes faced by HIV- infected individuals receiving HAART, they can also experience psychological responses such as agitation, confusion, anxiety, nightmares, mania and depression^25^. The finding that the subjects in the study group showed significant decrease in depression scores while those in the control group did not implies that six weeks aerobic exercise conducted for 30 min three times a week can lead to an appreciable improvement in the depressive symptoms. Hence aerobic exercise is important in reducing or preventing depression in people living with HIV. This result is consistent with several other studies. Belfield et al.^26^ revealed a significant decrease in depression after nine weeks of aerobic exercise in psychiatric patients. Schwandt et al.^27^ reported fewer symptoms of depression in individuals with traumatic brain injury after twelve weeks of aerobic exercise program. Mild to moderate aerobic exercise was effective in reducing depressive symptoms among breast cancer survivors^28^. Exercise training may be considered an alternative to antidepressants for the treatment of depression in older persons^29^. A significant decrease in depressive symptoms was found in Iranian women after four weeks of aerobic exercise showing that short term aerobic exercise can also effectively reduce depression^30^.

The limitations of this study included carrying out the work for short period of time because longer time might have led to decreased compliance with appointments and increased number of subjects’ dropout. The sample size was also relatively small of which not every subject recruited for the study completed the research.

Based on the study findings, it is therefore recommended that supervised aerobic exercise should be included as a routine treatment in HIV/AIDS clinics. Physiotherapists who prescribe exercises for a wide range of conditions should be involved in management of individuals living with HIV/AIDS in order to prescribe and supervise aerobic exercise programs that will improve pulmonary functions, reduce respiratory symptoms and depression.

## Conclusions

A six weeks aerobic exercise intervention was able to significantly improve pulmonary functions, reduce respiratory symptoms and depression in people living with HIV.

## Acknowledgments

The authors acknowledge all the patients that participated in this study.

## Conflict of interest statement

The authors have no conflict of interest.
